# Phloem anatomy predicts berry sugar accumulation across 13 wine-grape cultivars

**DOI:** 10.3389/fpls.2024.1360381

**Published:** 2024-03-21

**Authors:** Ryan C. Stanfield, Elisabeth J. Forrestel, Kayla E. Elmendorf, Sophia B. Bagshaw, Megan K. Bartlett

**Affiliations:** ^1^ Department of Biological Sciences, California State University, Stanislaus, Turlock, CA, United States; ^2^ Department of Viticulture & Enology, University of California Davis, Davis, CA, United States

**Keywords:** phloem area, Brix accumulation, berry ripening, climate adaptation, viticulture, sieve plates

## Abstract

**Introduction:**

Climate change is impacting the wine industry by accelerating ripening processes due to warming temperatures, especially in areas of significant grape production like California. Increasing temperatures accelerate the rate of sugar accumulation (measured in ⁰Brix) in grapes, however this presents a problem to wine makers as flavor profiles may need more time to develop properly. To alleviate the mismatch between sugar accumulation and flavor compounds, growers may sync vine cultivars with climates that are most amenable to their distinct growing conditions. However, the traits which control such cultivar specific climate adaptation, especially for ⁰Brix accumulation rate, are poorly understood. Recent studies have shown that higher rates of fruit development and sugar accumulation are predicted by larger phloem areas in different organs of the plant.

**Methods:**

Here we test this phloem area hypothesis using a common garden experiment in the Central Valley of Northern California using 18 cultivars of the common grapevine (*Vitis vinifera*) and assess the grape berry sugar accumulation rates as a function of phloem area in leaf and grape organs.

**Results:**

We find that phloem area in the leaf petiole organ as well as the berry pedicel is a significant predictor of ⁰Brix accumulation rate across 13 cultivars and that grapes from warm climates overall have larger phloem areas than those from hot climates. In contrast, other physiological traits such as photosynthetic assimilation and leaf water potential did not predict berry accumulation rates.

**Discussion:**

As hot climate cultivars have lower phloem areas which would slow down brix accumulation, growers may have inadvertently been selecting this trait to align flavor development with sugar accumulation across the common cultivars tested. This work highlights a new trait that can be easily phenotyped (i.e., petiole phloem area) and be used for growers to match cultivar more accurately with the temperature specific climate conditions of a growing region to obtain satisfactory sugar accumulation and flavor profiles.

## Introduction

Grapes are the most valuable fruit crop in the United States, valued at over $6.5 billion annually (Cheon et al., 2020), but climate change is projected to reduce grape production and quality (Jones et al., 2005; Roehrdanz and Hannah, 2016). Climate affects grape quality by impacting the concentration of sugars, organic acids, and secondary compounds (Jones et al., 2005; Conde et al., 2007; Palliotti et al., 2013; Torregrosa et al., 2017). The climatic conditions producing the highest quality wine cause the berries to reach optimal ratios between sugar and acid concentrations and maximum concentrations of pigment, aroma, and flavor compounds simultaneously (Gladstones, 2011). Hot temperatures accelerate sugar accumulation, forcing growers to harvest earlier, before berries reach optimal flavor development, to avoid the high alcohol content and insipid wine flavor from excessive sugar to acid ratios (Bigard et al., 2018; Delrot et al., 2020). Harvest dates have shifted earlier historically, and climate models predict further acceleration of ripening (Webb et al., 2007; De Orduna, 2010; Delrot et al., 2020). Growers can partly compensate through management practices, such as trimming canopies or using shade clothes to reduce the ratio of sugar supply to demand (Pastore et al., 2011; Parker et al., 2020; Aru et al., 2022; Faralli et al., 2022), though these practices are costly and increasingly ineffective in the face of climate change (Delrot et al., 2020). Planting existing cultivars or developing new cultivars with slower sugar accumulation are promising alternative strategies to mitigate these climate change impacts, but these efforts have been hindered by uncertainty around the plant traits controlling sugar accumulation (Bigard et al., 2018; Delrot et al., 2020; Gashu et al., 2020; Suter et al., 2021).

Grape cultivars vary in berry maturation and sugar accumulation rates, and in their response to abiotic stress, but the main anatomical and physiological mechanisms driving these differences remain unknown (Destrac-Irvine and Van Leeuwen, 2016; Vivin et al., 2017; Suter et al., 2021). Multiple physiological processes influence berry sugar accumulation and its responses to climate, including photosynthesis, long-distance sugar transport, and local transport and metabolism in the berries (Matthews and Shackel, 2005; Osorio et al., 2014; Savage et al., 2015; Nam et al., 2022). However, the relative importance of these factors in regulating sugar concentrations and fruit growth is debated (Savage et al., 2015). Photosynthetic responses to heat and water stress could impact cultivar differences in accumulation rates by affecting the sugar supply for ripening (Gambetta et al., 2020). Further, sugar is transported from the photosynthesizing leaves to the berries through the sugar-conducting vascular tissue – the phloem. At the onset of ripening (veraison), the berries significantly accelerate sugar accumulation by initiating active sugar unloading from the phloem, making the phloem the primary pathway for water and resource influx into the berries (Matthews and Shackel, 2005; Zhang et al., 2006). The importance of phloem transport to ripening suggests that phloem traits could be important drivers of cultivar differences in sugar accumulation, and that modifying phloem traits to slow sugar accumulation under hot conditions could help mitigate the impacts of climate change on wine quality. However, the main traits controlling sugar accumulation in grape remain unclear (Suter et al., 2021).

The rate of phloem transport is determined by both the hydraulic resistance to the flow of sugar sap, and the activity and kinetics of water and sugar transporters in the sources, sinks, and along the transport pathway (Stanfield et al., 2017, 2019). Modeling studies suggest that increasing the hydraulic resistance of the phloem reduces sugar export to the sinks (Stanfield and Bartlett, 2022). Therefore, selecting grape cultivars with lower total phloem conductance could decelerate sugar accumulation and improve the synchronization of sugar accumulation with flavor development under hotter conditions. However, a higher hydraulic resistance can make the phloem more susceptible to declines or even complete failures in transport under severe water stress (Sevanto, 2018; Stanfield and Bartlett, 2022). Thus, we expect cultivars that produce high-quality wine in hot, dry conditions to exhibit phloem hydraulic resistances that slow berry sugar accumulation while avoiding phloem failure. The phloem transport pathway is composed of individual sugar-conducting cells (sieve elements) with porous end walls (sieve plates) stacked to form conduits (sieve tubes). The anatomy of the transport pathway, including the total cross-sectional area of sieve tubes in plant organs, lumen area of individual sieve tubes, and porosity of the sieve plates, significantly impacts pathway resistance (Esau et al., 1957, Geiger et al., 1969; Grange and Peel, 1975; Lush, 1976; Milburn and Kallarackal, 1984; Mullendore et al., 2010; Stanfield et al., 2019; Ray and Savage, 2020). Plants with a greater cross-sectional area dedicated to phloem (Hölttä et al., 2006), sieve tubes with wider lumen areas (Jensen et al., 2009), and larger and more abundant pores in the sieve plates are expected to have a lower hydraulic resistance (i.e., higher conductance) (Mullendore et al., 2010). Total phloem cross-sectional area in the shoots has been found to vary between several grape cultivars (Esau, 1948), and a greater cross-sectional phloem area has been linked to faster sugar accumulation in the fruit in other crop species (Savage et al., 2015; Nam et al., 2022). However, the variation of phloem structural traits across cultivars adapted to a diverse range of climatic conditions and the relationship of these traits to sugar accumulation is largely unknown for grapevines. Establishing these anatomical links could allow breeders to modify sugar accumulation by selecting for phloem traits, instead of management practices that can negatively impact the fruit zone environment or yield (e.g., modifying crop load or canopy area).

In this study, we used a common garden experiment to evaluate the links between phloem anatomy and sugar accumulation across 18 winegrape cultivars typically grown in climatically diverse grape-growing regions. We assessed phloem and xylem vascular anatomy in leaf petioles and midveins and berry pedicels, to capture hydraulic resistance along the long-distance transport pathway. We also measured maximum berry sugar accumulation rates in the post-veraison ripening period to capture the greatest capacity for sugar transport (Savage et al., 2015; Nam et al., 2022). We predicted that traits that reduce hydraulic resistance, including larger total cross-sectional phloem areas, larger mean lumen areas for individual sieve tubes, and more porous sieve plates would increase maximum sugar accumulation rates. We also predicted that cultivars typically grown in hotter wine regions would have traits that increase hydraulic resistance, as an adaptation to increase wine quality by reducing the rate of sugar accumulation. In addition, we measured photosynthesis and vine water stress to compare the impacts of phloem anatomy, vine carbon supply, and vine water status on sugar accumulation rates. Overall, our goals were to determine the most influential traits for sugar accumulation in grape berries and evaluate the role of phloem anatomy in adapting grape cultivars to a wide range of different climates.

## Materials and methods

### Study site and plant material

Berry chemistry, anatomy and physiology were measured in summer 2020 for 18 grape cultivars (*Vitis vinifera* subsp*. vinifera* L.) established in an experimental vineyard on the University of California, Davis campus (38.53 N, -121.75 W) (**Table 1**). There were 13 red-fruited and 5 white-fruited cultivars. Further, 9 cultivars were classified as hot-climate, 7 as warm-climate and 2 as temperate-climate, using the definitions from Anderson & Nelgen (2020). Anderson & Nelgen sorted the major wine-growing regions worldwide into climate categories based on mean temperature over the growing season (i.e., temperate-climate = 15 - 17°C, warm = 17 - 19°C, and hot >19°C). Cultivars were then placed into their respective climate category based upon the highest proportion of bearing area grown in a particular climate category as of 2020. This proportion of land area devoted to growing a particular cultivar worldwide was taken as a thermal requirement, genotypically driven, to match sugar accumulation with a region’s climate.

**Table 1:** Sampled cultivars by Growing Season average Temperature (GST) globally and classified by the percentage of total land area of each cultivar grown in each of four climate categories; cool (<15°C), temperate (15-17°C), warm (17-19°C) and hot (>19°C) (based upon Table 77, Anderson and Nelgen, 2020).

Variety	Area (ha)	Color	GST (°C)	Cool (%)	Temp (%)	Warm (%)	Hot (%)	Climate Category
Zinfandel (*Tribidrag)*	33649	Red	19.6	0	3	15	82	Hot
Syrah	181185	Red	19.4	0	4	46	51	Hot
Sangiovese	73464	Red	19.5	0	0	12	88	Hot
Tempranillo	219379	Red	18.8	0	25	19	56	Hot
Aglianico	9734	Red	19.1	0	0	27	73	Hot
Montepulciano	32935	Red	19.5	0	0	17	83	Hot
Fiano	95	White	19.9	0	2	42	56	Hot
Verdelho	1156	White	20.8	0	1	13	86	Hot
Mourvèdre (Monastrell)	51930	Red	20.8	0	0	11	89	Hot
Riesling	59805	White	16.2	23	41	33	3	Temp.
Pinot Noir	105480	Red	16.4	28	37	28	7	Temp.
Chardonnay	201649	White	18.2	7	23	39	31	Warm
Merlot	266440	Red	18.2	1	33	44	23	Warm
Nebbiolo	7997	Red	18.4	1	1	77	22	Warm
Carignan (Mazuelo)	47312	Red	19.4	0	1	58	41	Warm
Barbera	17824	Red	18.9	0	0	69	31	Warm
Cabernet Sauvignon	310671	Red	18.5	0	14	57	29	Warm
Sauvignon Blanc	124700	White	17.7	2	35	45	19	Warm

Cultivars were categorized into their respective groups if the majority percentage of their land area occurred in that climate category. For anatomical sampling of pedicel, and leaf organs, each cultivar was sampled twice (n = 2).

Plants were growing as mature (9-year-old) vines, grafted to the same rootstock (420A), and trained to a bilateral, spur-pruned, vertical shoot-positioned trellising system with a North-South row orientation (*N* = 2 vines per cultivar). Cultivars were divided between two adjacent vineyard blocks (**Supplementary Table S1**). Davis is considered a hot, dry site for winegrowing, with campus weather stations reporting a decadal average mean annual precipitation of 436 mm and mean growing season temperature of 19.8°C (Davis CIMIS station, 38°32’8N/121°46’35W, 2009-2019, https://cimis.water.ca.gov). Our study period (July 24 – September 11, 2020) was exceptionally hot, with mean daily and mean maximum daily temperatures ranging from 22.8-24.5°C and 32.6-34.4°C, respectively, partly due to the anomalous August 16 – 18 heatwave (**Supplementary Figure S1**). Over the study period, vines were drip-irrigated weekly at 50% replacement of vineyard evapotranspiration, which was estimated from the reference evapotranspiration reported by the campus weather status and published crop coefficients for this trellising system and vine × row spacing (i.e., 50 – 100 liters per vine per week, see Williams et al., 2022).

### Brix measurement

Berries were sampled at regular intervals defined by Brix values from 50% veraison to harvest (July 22, 24, and 30, August 6, 22, and 24, and September 2). For each cultivar, 30 berries per vine were collected from different parts of the cluster and both sides of the vine from 2 – 6 vines (n =2-6). Berries of each replicate were crushed, and the grape juice obtained was centrifuged at 4200 rpm for five minutes. Next, each juice sample was analyzed for TSS (Total Soluble Solids) using a refractometer Sper Scientific 30051 (Sper Scientific LTD, Scottsdale, AZ, USA), pH with an Orion Star A211 pH meter (Thermo Fisher Scientific Inc., Waltham, MA, USA), and titratable acidity by titration with 0.1 N NaOH (VWR International, Radnor, PA, USA) with an end point at pH 8.2 (Iland, 2004).

### Anatomical sampling

**Figure 1:**
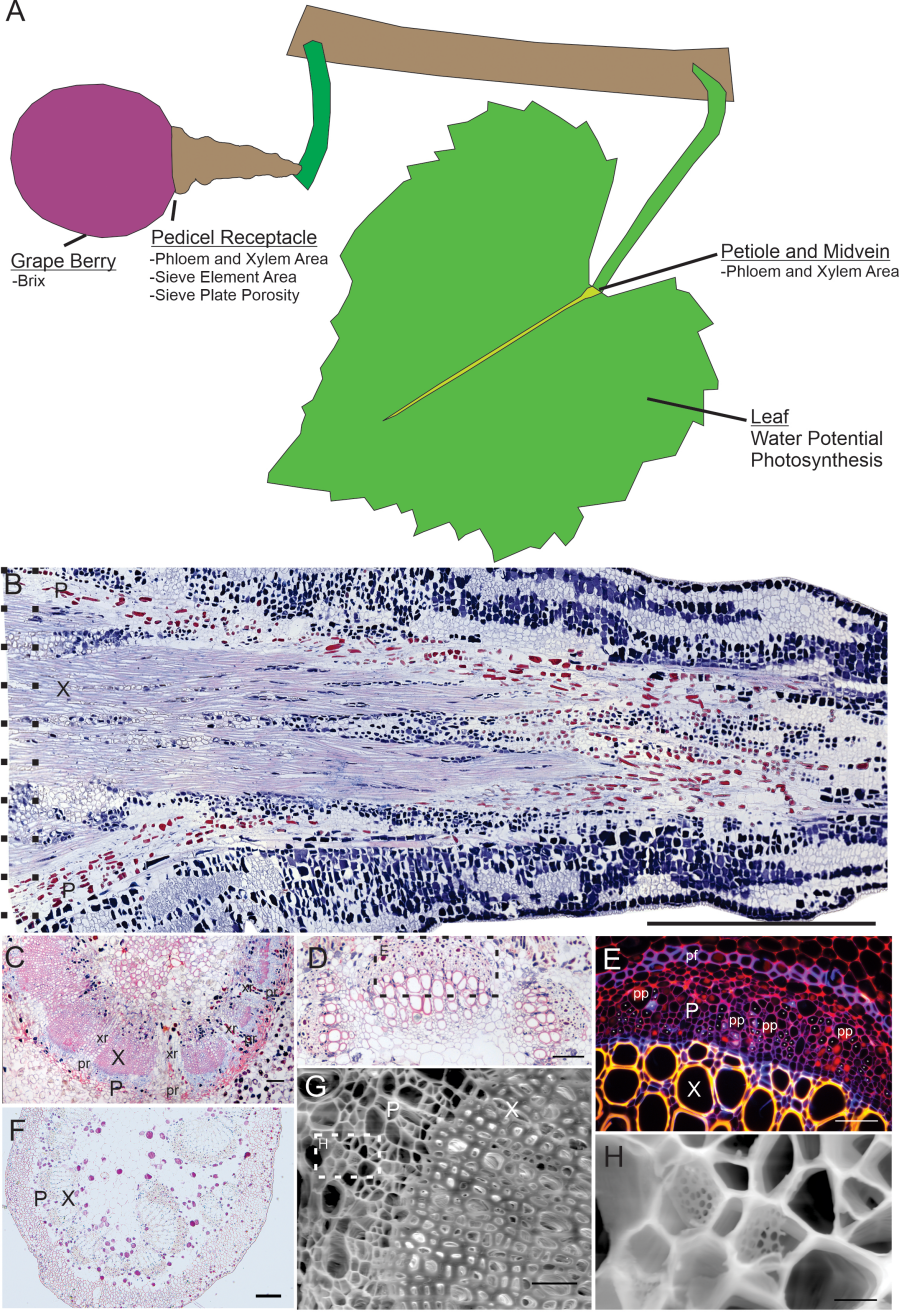
Overview of organs sampled, and representative anatomical sections surveyed. **(A)** Overview diagram of the organs sampled for chemistry (grape berry) and organs sectioned for anatomy (pedicel, petiole, midvein), as well as leaf physiology data. **(B)** Longitudinal section of grape berry pedicel showing phloem area (P) and xylem area (X). Dotted lines indicate the approximate area of the pedicel receptacle where cross sectional areas were measured. Scale = 1mm. **(C)** Cross section of pedicel showing xylem, phloem, xylem rays (xr) and phloem rays (pr). Scale = 100µm. **(D)** Cross section of a leaf midvein. Scale = 100µm. **(E)** Inset from panel D showing magnified area under fluorescent light (G/R filter cube), labeling xylem, phloem, phloem fibers (pf) and phloem parenchyma (pp). Asterisks indicate location of sieve elements based upon callose fluorescence signature. Scale = 100µm. **(F)** Cross section of petiole showing xylem and phloem. Scale = 100µm. **(G)** SEM image of pedicel showing xylem and phloem. Scale = 50µm. **(H)** Magnified inset from panel G showing example sieve plates. Scale = 5µm. All shown sections taken from Syrah.

Leaves and berries were sampled to measure petiole, midvein, and pedicel anatomy in the morning (7 – 11AM) on three days at the end of the growing season (September 9 - 11, 2020). Two berries and leaves per vine were excised with a razor blade. Leaf position was standardized as the 6^th^ leaf from the shoot apex, to capture the most photosynthetically active leaves. Two leaf and one berry sample per vine were then prepared for light microscopy, and the other berry sample was prepared for scanning electron microscopy. For light microscopy, a 1-cm segment of leaf petiole and lamina and the entire pedicel of the berry (**Figure 1A**) were immediately excised and placed into a vial of chilled Formalin-Acetic Acid (FAA). Vials were put on ice and refrigerated at 4°C for at least 24 hours before further processing.

For scanning electron microscopy, pedicels were immediately flash-frozen and immersed in liquid nitrogen for 1-min and placed into a chilled micro-centrifuge tube of 100% ethanol, then the tube was immersed in liquid nitrogen until the ethanol congealed (Mullendore, 2019). Samples were then immediately placed on ice and stored in a -20°C freezer for at least 24 hours before further processing.

### Light microscopy

After 7 days in FAA, the light microscopy samples were soaked in 50% ethanol for 5 mins and then stored in 70% ethanol in preparation for paraffin embedding. Samples were first infiltrated with paraffin by using an Autotechnicon Tissue Processor to treat samples with the following sequence of solutions: 70%, 85%, 95%, 100% (x2) ethanol, 1 ethanol:1 toluene, 100% toluene (x2), and paraffin wax (x2), each for 1 hour. The infiltrated samples were then embedded into paraffin blocks with a Leica Histo-Embedder (Leica Microsystems, Wetzlar, Germany), and allowed to cool. A rotary microtome was then used to make 7µm-thick cross-sections for leaf laminas, petioles, and berry pedicels. Pedicel cross-sections were sampled from the receptacle (**Figure 1B**, longitudinal pedicel section between dashed lines) and petiole and midvein cross-sections were sampled near the interface of the lamina and petiole. After the cross-sections were imaged, pedicels for four cultivars (i.e., Syrah, Barbera, Fiano and Verdelho) were remelted from their wax molds, oriented longitudinally and sectioned again at 7µm to obtain sieve element lengths. Sections were stained using a 1% aniline blue and 1% safranin solution following a modified staining procedure (Clark and Biological Stain Commission, 1981). Sections were then viewed under brightfield or florescence (using the G/R filter cube) microscopy using a Leica DM4000B microscope and a DFC7000T digital camera (Leica Microsystems, Wetzlar, Germany).

Each pedicel (**Figures 1B, C**), midvein (**Figures 1D, E**), and petiole (**Figure 1F**) section was then measured for total phloem and xylem cross-sectional area using ImageJ software, by manually selecting relevant tissue areas. Vascular tissue (phloem or xylem) in longitudinal sections (**Figure 1B**) and cross sections (**Figures 1C–F**) was identified by cell size and/or stain color. Safranin stained the secondary cell walls of the xylem red and phloem cell walls were stained blue by aniline blue. The phloem area measurements included sieve tubes (**Figure 1E**, asterisks) and phloem fibers (**Figure 1E**, pf) and parenchyma (**Figure 1E**, pp), and xylem area measurements included xylem vessels, fibers, and parenchyma. Xylem (**Figure 1C**, xr) and phloem (**Figure 1C**, pr) rays greater than 4 cell layers thick were excluded. For the four cultivars measured for sieve element length, we also calculated total phloem resistance (Equation 1), using the formula from Liesche et al. (2017):


(1) $$R_{SE}=\frac{1}{N_{p}}\left(\frac{3\eta }{r_{p}^{3}}A+\frac{8\eta l_{p}}{\pi r_{p}^{4}}B\right)+\;\frac{8\eta l}{\pi r^{4}}$$

where *N_p_* is the number of pores per plate, r_p_ = sieve pore radius, *l* = sieve element length, *l_p_* = sieve plate thickness and r = sieve element radius, η the viscosity of the phloem sap (2mPa*s), and A and B are variables which account for the variation in sieve plate pore diameter (see Liesche et al., 2017 for more details).

### Scanning electron microscopy

The pedicel electron microscopy samples were processed following Mullendore (2019). Briefly, samples were thawed at room temperature, washed in DI water, and cut into 1 mm cross sections with a fresh double-sided razor blade. Sections were then transferred to 1.5 ml of 0.15% Proteinase K (p-protein digestion) solution and mixed at 55°C and 300 RPM rotation for 14 days with an Eppendorf Thermomixer (Eppendorf North America, Framingham, MA, USA). Samples were then washed in DI water and placed into an 0.1% amylase solution (starch digestion) for 24 hours at 50°C. Samples were then washed in DI water again, lyophilized overnight, mounted on aluminum stubs, and viewed under a Field Emission Scanning Electron Microscope (FESEM; Thermo Fisher Quattro S, Waltham, MA, USA). Sieve plates were viewed under low vacuum (50 pa), 20-KV of accelerating voltage and a spot size of 2.5.

Mean sieve element area and sieve plate porosity were measured by manually tracing individual sieve elements (**Figure 1G, H**) and sieve plates and pores with ImageJ software. Sieve element area was calculated by identifying 1 – 3 sieve plates and 3 – 11 images per vine. Sieve element area measurements included the cell wall area. Sieve plate porosity (Equation 2) was calculated as:


(2) $$Porosity=\frac{\Sigma \;Sieve\;Plate\;Pore\;Area}{Total\;Sieve\;Plate\;Area}$$

### Water potential and photosynthesis measurements

Leaf water potentials (one per vine) were measured at pre-dawn and midday every one to two weeks from July 23 to September 3. Predawn, and midday stem water potentials (Ψ_stem_) and leaf water potentials (Ψ_leaf_) were taken using Scholander pressure chambers (PMS Instrument Company, Albany, OR, USA). Predawn water potentials were collected prior to sunrise (4 – 5 am). Midday Ψ_stem_ and Ψ_leaf_ were carried out between noon and 2 pm, and midday Ψ_leaf_ measurements were performed on the same leaf as the gas exchange measurements. Cut leaves were kept in opaque Whirl-Pak (WHIRL-PAK, Madison, WI, USA) bags, and placed on ice and brought to the laboratory for measurement. Leaf water potentials were measured using a Model 600D Pressure Chamber (PMS Instrument CO, Albany, OR, USA).

Photosynthesis data was collected weekly from July 23 to September 3 using a LI-COR 6800 infrared gas analyzer (LI-COR Biosciences, Lincoln, NE, USA). Midday gas exchange measurements were taken between noon and 2 pm, and PAR was set at the 1600 µmol m^-2^ s^-1^. Air flow was set at 500 µmol m^-2^ s^-1^, reference CO_2_ concentration established at 400 µmol m^-2^ s^-1^, and chamber humidity was set to 50%.

### Statistical analyses

**Figure 2:**
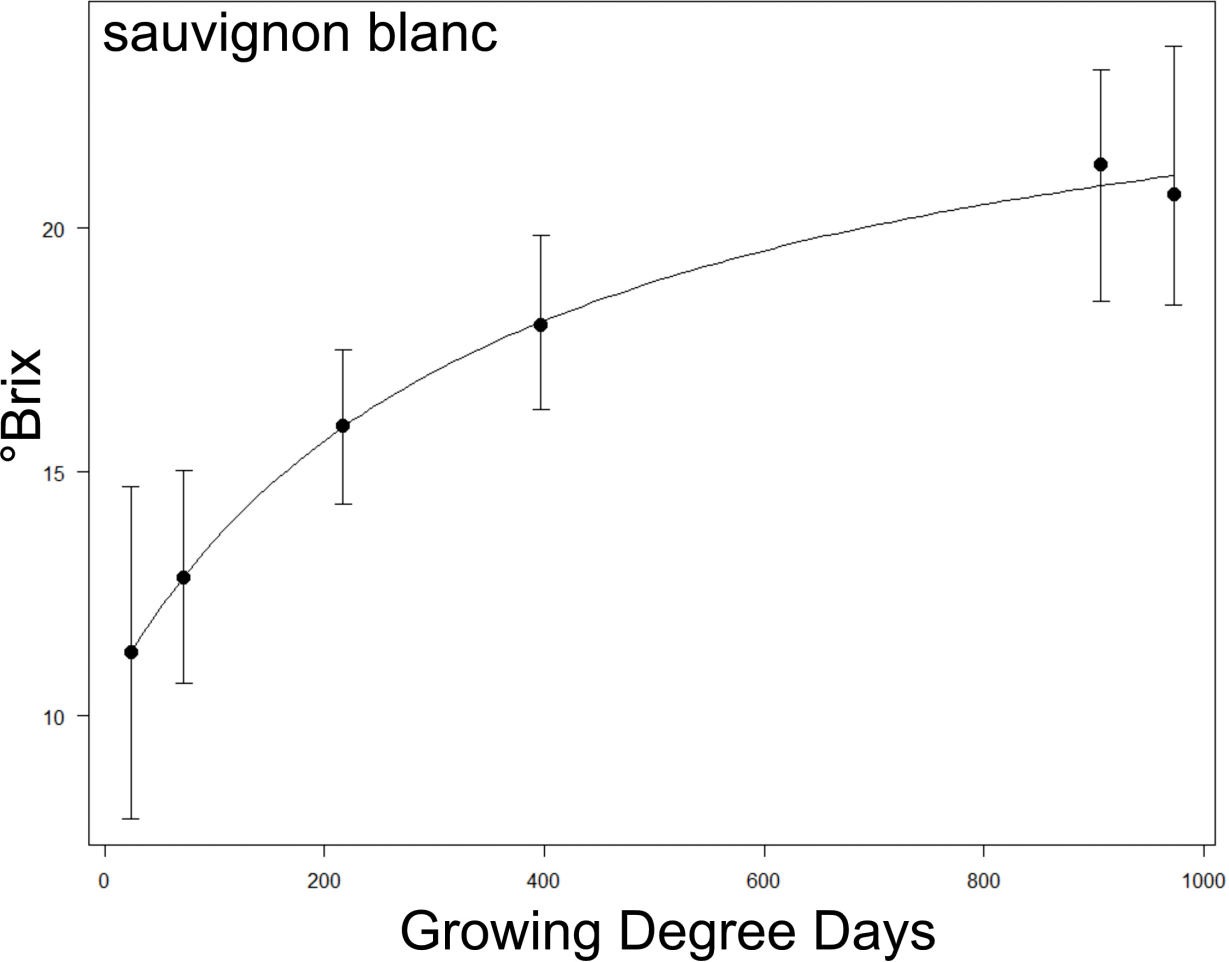
Example of a °Brix sugar accumulation curve over Growing Degree Days (GDD) taken from Sauvignon Blanc. The relationship between the two parameters was fitted to a logistic function, and the maximum °Brix accumulation rate was extracted via the maximum steepness of the fitted curve (see methods). See **Supplementary Figure S2** for curves of all cultivars.


First, we tested correlations between the anatomy traits and maximum sugar accumulation rates across cultivars. The maximum sugar accumulation rate for each cultivar was calculated from a logistic function (Equation 3), similar to methods of Sadras et al. (2008). We fitted a 4-parameter logistic function to estimate °Brix (S) as a function of Growing Degree Day (x; see calculation below, and **Figure 2** for an example):

(3) $$\text{S }=\text{Smin}+\frac{\text{ Smax }-\text{ Smin}}{\left(1\;+\;exp^{(\text{b}\left(\text{x }-\text{ x}0\right)}\right)}\;$$

where Smin is the minimum °Brix at the bottom of the curve, Smax is the maximum °Brix at the asymptote, b is the initial slope of the curve and x0 is the °Brix at half maximum. The maximum brix accumulation rate (Equation 4) was calculated as:


(4) $$=\frac{Smax}{transition\;width}$$

where transition width is the number of Growing Degree Days between the 1^st^ and 3^rd^ interquartile range of growing degree days encompassed by the logistic model. Essentially, this provides a slope at the steepest part of the curve to estimate the Maximum Δ °Brix/Growing Degree Day. See **Supplementary Figure S2** for the accumulation curves of each cultivar. Note that Zinfandel, Aglianico, Pinot Noir, Chardonnay and Sauvignon Blanc were excluded from the analysis as they did not fit the logistic model. Logistic curves were fitted to each cultivar using the LL.4 function DRC package in R (https://cran.r-project.org/web/packages/drc/drc.pdf).

Growing Degree Days (GDD) (Equation 5) was used instead of time, since sugar accumulation is closely related to heat accumulation (Greer and Weedon, 2014). GDD was calculated from the beginning of the berry chemistry sampling period (July 22) as:


(5) $$GDD={\;}^{\sum }i=1\left[\frac{T_{MAX}+T_{MIN}}{2}\right]-T_{BASE}$$

where T_MAX_ and T_MIN_ are the daily maximum and minimum temperatures, respectively, and T_BASE_ is a minimum threshold growing temperature, which was set to the historic default for grapevine, 10°C (McMaster and Wilhelm, 1997; Zapata et al., 2015). Simple linear regressions were then used to test correlations between maximum °Brix accumulation rates and leaf lamina, petiole, and pedicel phloem and xylem cross-sectional area and mean sieve element area and sieve plate porosity for the pedicels. Two cultivars (Pinot Noir and Sauvignon Blanc) were excluded due to sample quality being too poor to establish reliable anatomical measurements. We also examined relationships between xylem and phloem anatomy by using linear regression to test correlations between xylem and phloem cross-sectional area.

Second, we used two-way ANOVAs to test for significant differences in the phloem and xylem anatomy traits between climate and color groupings. We excluded the temperate-climate group from this analysis due to the small sample size. Multiple comparisons were made using the Tukey HSD test.

Third, we used multiple linear regressions to test whether photosynthetic assimilation or minimum mid-day leaf water potentials improved the prediction of maximum °Brix accumulation (see **Table 2** for list of models). Critical values were set at the P< 0.05 significance level and all statistics were performed in R Studio version 2022.02.3 Build 492.

**Table 2:** Multiple regression models using combinations of phloem anatomy, photosynthesis (A) at maximum brix accumulation rate and mid-day water potential (WP) at maximum brix accumulation rate as predictors for the maximum brix accumulation rate (Brix).

Model #	Model	r^2^	p-value	AICc
1	Brix = A Brix+WP Brix+phloem midvein+phloem petiole+phloem pedicel	0.66	0.113	-29.6
2	Brix = phloem petiole + phloem pedicel + A brix + Wp brix	0.537	0.144	-36.0
**3**	**Brix = phloem midvein+phloem petiole+phloem pedicel**	**0.576**	**0.044**	**-44.5**
**4**	**Brix = phloem petiole+phloem pedicel**	**0.481**	**0.038**	**-47.5**
**5**	**Brix = phloem pedicel**	**0.471**	**0.007**	**-56.8**
**6**	**Brix = phloem petiole**	**0.315**	**0.046**	**-34.8**
7	Brix = phloem midvein	0.01	0.272	-49.4
8	Brix = A Brix + WP Brix	0.232	0.235	-47.6
9	Brix = A Brix	0.028	0.569	-48.3
10	Brix = WP Brix	0.052	0.433	-48.7
11	Brix = phloem midvein+phloem petiole+phloem pedicel+A Brix	0.617	0.075	-38.4
12	Brix = phloem midvein+phloem petiole+phloem pedicel+WP Brix	0.576	0.107	-37.1
**13**	**Brix = phloem petiole+phloem pedicel+Max Brix**	**0.585**	**0.04**	**-44.8**
14	Brix = phloem petiole+phloem pedicel+A Brix	0.485	0.1	-42.0
15	Brix = phloem midvein+phloem petiole+phloem pedicel+A Brix	0.577	0.11	-37.0
16	Brix = phloem petiole+phloem pedicel+A Brix +WP Brix	0.494	0.195	-34.8
17	Brix = phloem petiole+phloem pedicel+WP Brix	0.494	0.092	-42.2
18	Brix = phloem petiole+WP Brix	0.355	0.112	-44.7
19	Brix = phloem petiole+A Brix	0.321	0.145	-44.0
20	Brix = phloem petiole+A Brix +WP Brix	0.36	0.239	-39.2

Also included are Aikake Information Criterion corrected for small sample size (AICc) with lower values associated with more likely models.Bold values show significant models of P < 0.05.

## Results

### Vascular area in petioles and pedicels significantly predicted maximum °Brix accumulation rates

**Figure 3:**
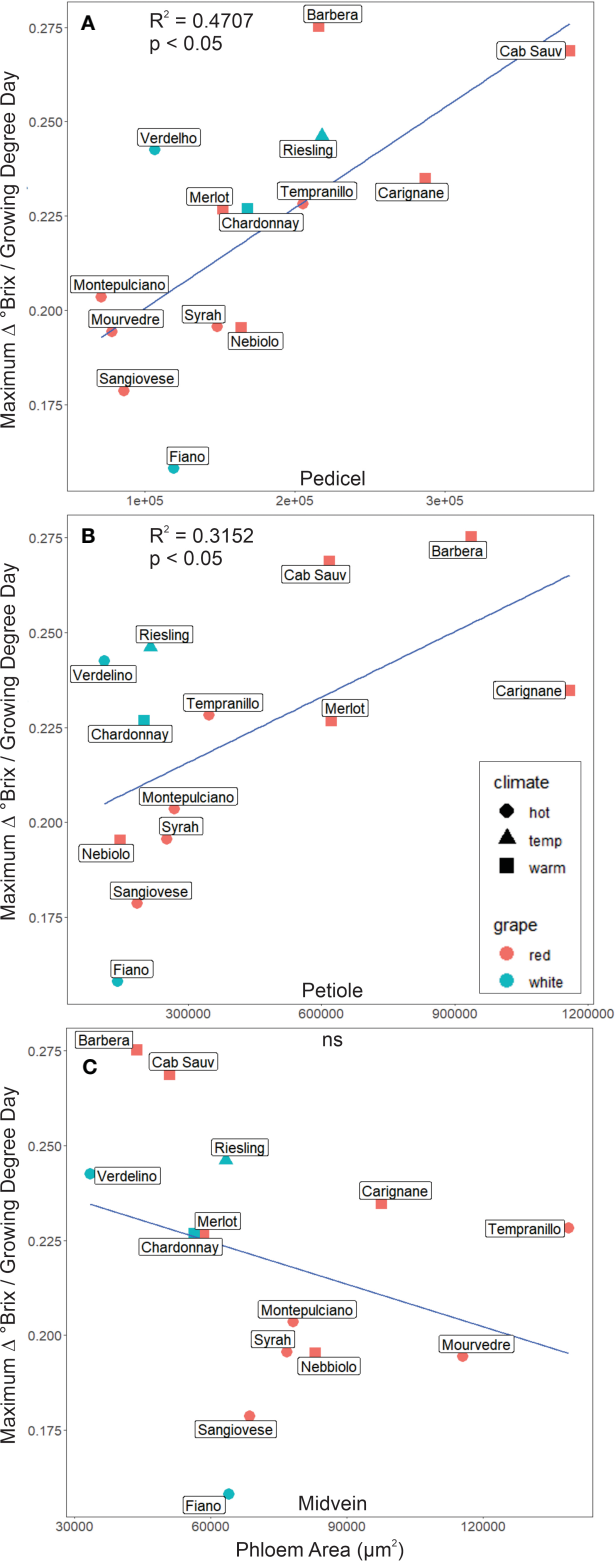
The maximum rate of change in °Brix as a function of phloem area for different cultivars sampled. **(A)** Phloem area was a significant predictor of °Brix accumulation rate in pedicels in addition to **(B)** petioles. In contrast, phloem area in **(C)** midveins was not a significant (ns) predictor of °Brix accumulation rate.

Maximum sugar accumulation rates were significantly correlated with the total cross-sectional phloem area in the berry pedicels (r^2^ = 0.47, p< 0.05, *N* = 14, **Figure 3A**) and leaf petioles (r^2^ = 0.32, p< 0.05, *N* = 13, **Figure 3B**) but not the midveins (r^2^< 0.1, p ≥ 0.05, *N* = 14, **Figure 3C**). Maximum sugar accumulation was faster for the cultivars with larger cross-sectional phloem areas (**Figures 3A, B**). For the petioles, this relationship was driven by the allocation of cross-sectional area to the phloem, since maximum sugar accumulation rates were significantly correlated with the ratio of phloem to total petiole cross-sectional area (r^2^ = 0.48, p< 0.05, N = 12, **Supplementary Figure S3B**), but not with total petiole area (r^2^< 0.01, p ≥ 0.05, N = 12, **Supplementary Figure S4**).

**Figure 4:**
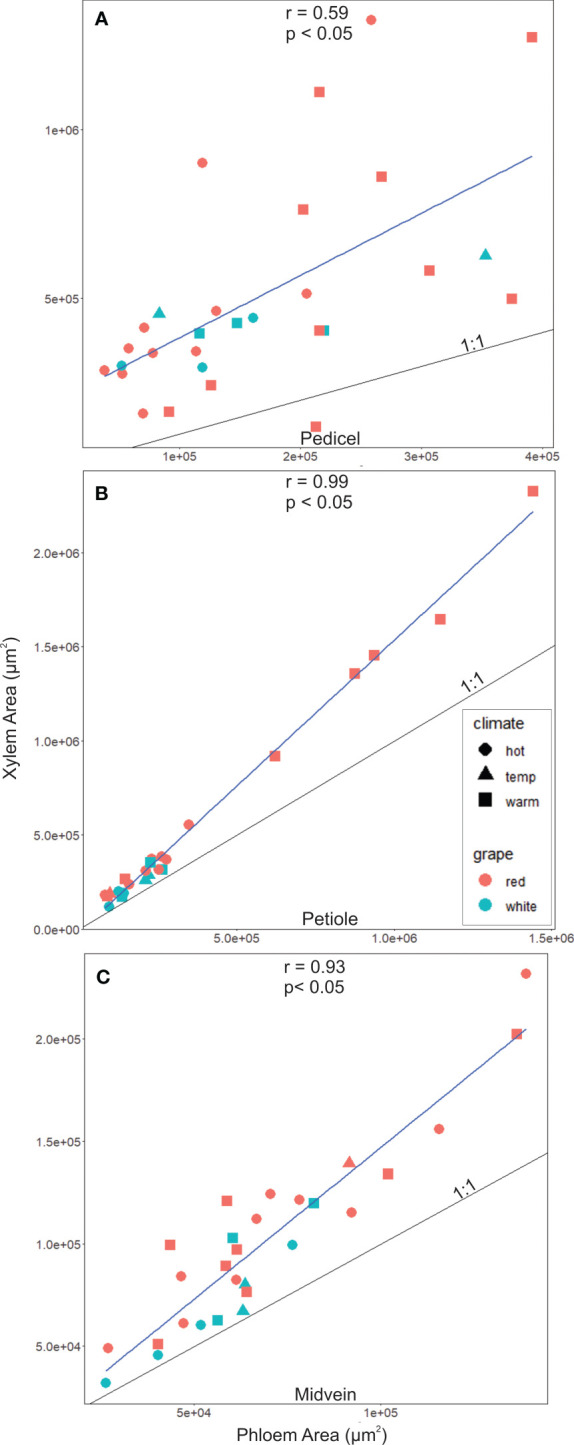
Xylem to phloem areas significantly correlated in **(A)** Pedicels, **(B)** Petioles and **(C)** midveins. In all organs, tissue was xylem dominated (i.e., points above the 1:1 line), however, this was much more the case in pedicels as opposed to petiole and midvein sections.

Xylem and phloem areas were closely correlated, making the relationships between maximum sugar accumulation rates and xylem area like those for phloem area. The correlations between phloem and xylem areas were significant and positive in the pedicel (R = 0.59, P< 0.05, N = 29), petiole (R = 0.99, P< 0.05, N = 25; **Figure 4B**), and midvein (R = 0.93, P< 0.05, N = 28, **Figure 4C**). In all organs, the cross-sectional area of vascular tissue was dominated by the xylem, and the xylem area was larger than the phloem in nearly all sampled sections (**Figures 4A–C**, points above black 1:1 line). Faster maximum sugar accumulation rates were not significantly related with larger xylem areas in the pedicels (r^2^ = 0.17, p ≥ 0.05, N = 14), petioles (r^2^ = 0.3, p ≥ 0.05, N = 14) and the midveins (r^2^ = 0.02, p ≥ 0.05, N= 14).

**Figure 5:**
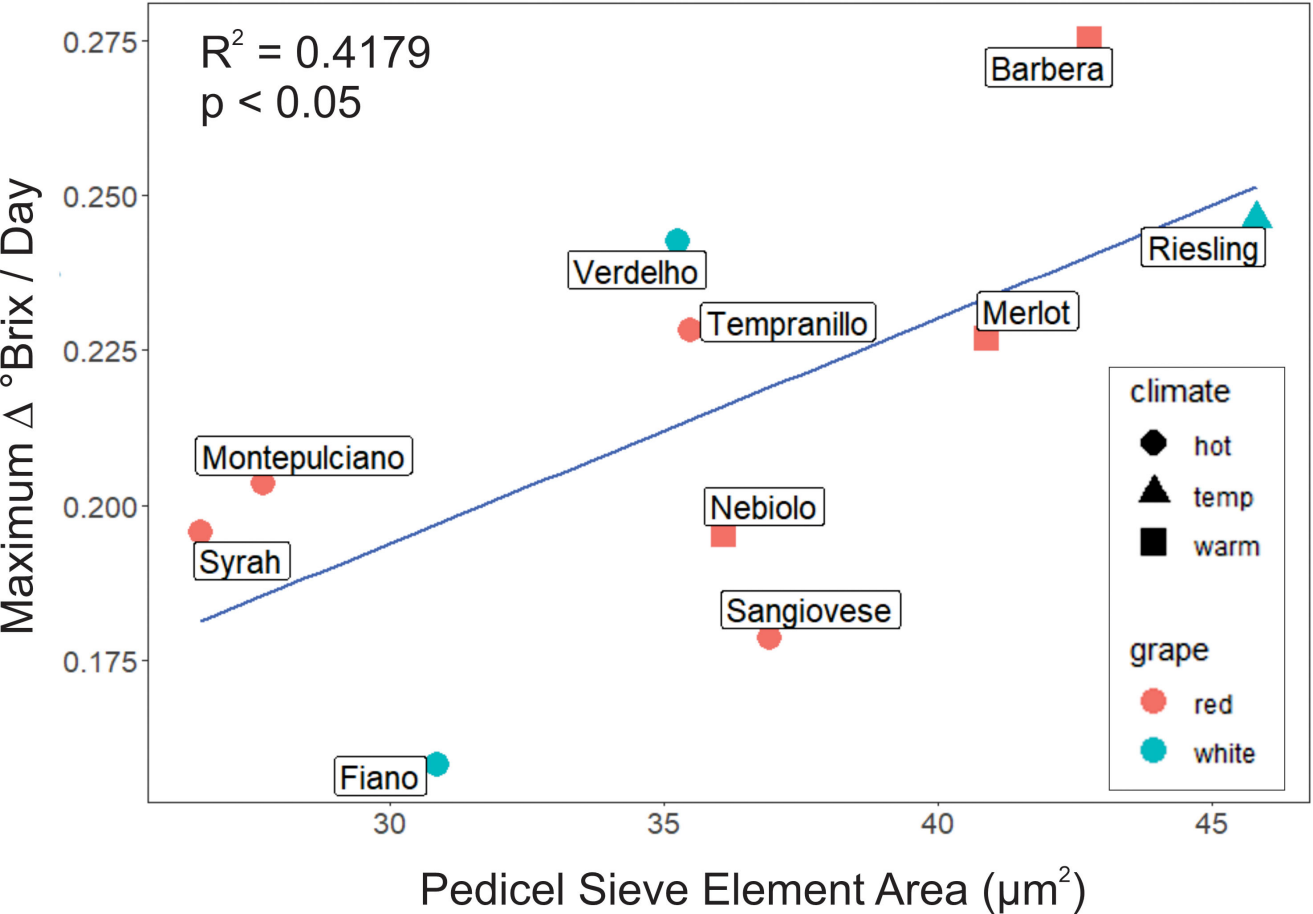
Mean area of sieve elements in the pedicel receptacle was a significant predictor of maximum °Brix accumulation rate, with larger sieve elements associated with higher accumulation rates.

However, maximum sugar accumulation rates were significantly correlated with mean sieve element area (r^2^ = 0.4179, p< 0.05, N = 10, **Figure 5**) but not sieve plate porosity in the pedicels (r^2^ = 0.03, p ≥ 0.05, N = 10).

### Phloem cross-sectional area was larger in warm- than hot-climate cultivars

**Figure 6:**
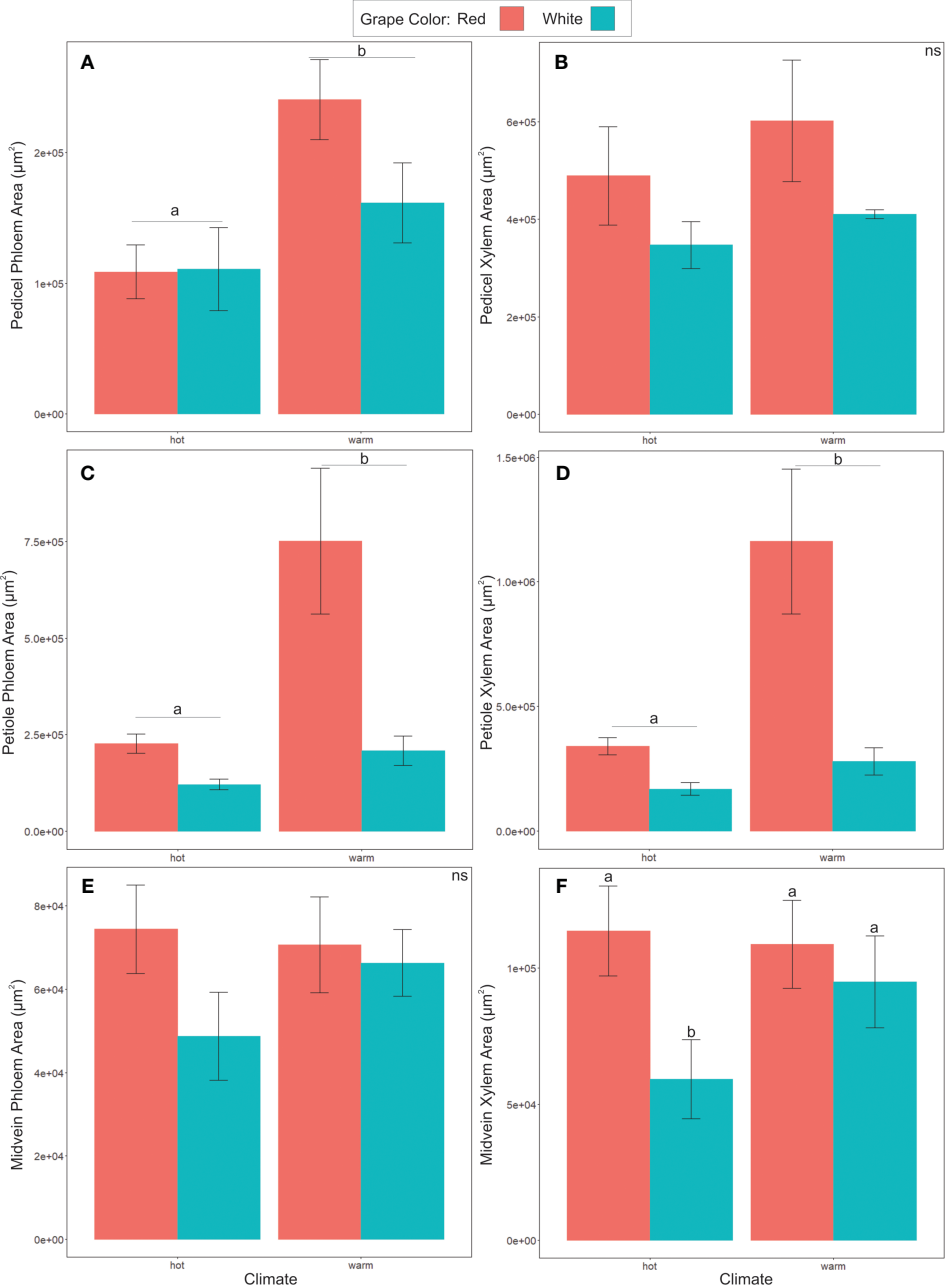
Comparison of xylem and phloem area, categorized by climate classification or grape color. **(A, B)** show phloem and xylem areas of pedicels, with phloem area of warm cultivars being significantly greater than hot cultivar grapes. **(C, D)** shows warm cultivar phloem and xylem petiole area significantly greater than hot cultivar grapes. **(E, F)** Midvein phloem and xylem area, showing significant differences between white and red grape xylem area within the hot cultivars only. Different letters indicate differences between groups. ns = not significantly different.

Phloem anatomy was significantly different between cultivars from different climate groups (**Figure 6**). The total phloem cross-sectional area was significantly larger in warm than hot-climate cultivars in the pedicels (ANOVA, F = 13.866, P< 0.05, N = 27) and petioles (F = 5.652, P<0.05, N = 22, **Figures 6A, C**) (See **Table 3** for ANOVA table). Conversely, phloem area was not significantly different between climate groups in the midvein (F = 0.005, P ≥ 0.05, N = 24). On average, total phloem area was 104% larger in the pedicels, 193% larger in the petioles, and 4% larger in the midveins in the warm- than hot-climate cultivars. Phloem area was also significantly larger for red than white grape cultivars in the petioles (F = 5.04, P< 0.05, N = 22), but not the pedicels (F = 1.084, P ≥ 0.05, N = 27) or midveins (F = 1.742, P ≥ 0.05, N = 24). Total organ cross-sectional area was not significantly different between climate or berry color groupings for any organ, signifying that the climate and color groups vary in vascular area and not overall organ size (P ≥ 0.05).

**Table 3:** ANOVA models used to test differences in phloem/xylem traits between grape color and climate grouping.

Model	F	P
Pedicel Models		
Xylem Cross Sectional Area		
Grape Color	1.173	P ≥ 0.05
Climate Type	0.603	P ≥ 0.05
Phloem Cross Sectional Area		
Grape Color	1.084	P ≥ 0.05
**Climate Type**	**13.866**	**P< 0.05**
**phloem:CS by Climate type**	**4.523**	**P< 0.05**
phloem:CS by Color	0.741	P ≥ 0.05
Petiole Models		
Xylem Cross Sectional Area		
**Grape Color**	**5.669**	**P< 0.05**
**Climate Type**	**9.638**	**P< 0.05**
Phloem Cross Sectional Area		
**Grape Color**	**5.04**	**P< 0.05**
**Climate Type**	**9.475**	**P< 0.05**
**phloem:CS by Climate Type**	**11.366**	**P< 0.05**
phloem:CS by Grape Color	3.255	P ≥ 0.05
Midvein Models		
Xylem Cross Sectional Area		
Grape Color	3.434	P ≥ 0.05
Climate Type	0.094	P ≥ 0.05
Phloem Cross Sectional Area		
Grape Color	1.742	P ≥ 0.05
Climate Type	0.005	P ≥ 0.05

Significant differences (P< 0.05) are shown in bold. Phloem : CS = phloem area to cross sectional ratio.

Xylem area was also significantly larger in warm- than hot-climate cultivars in the petioles (F = 9.638, P< 0.05, N=22), but not significantly different between climate groups in the pedicels or midveins (P ≥ 0.05) (**Figures 6B, D, F**). Xylem cross-sectional area was also significantly larger in red than white grape cultivars in the petioles (F = 4.92, P< 0.05, N=22) and midveins (F= 4.664, P< 0.05, N=25), though not the pedicels (**Figures 6B, D, F**).

**Figure 7:**
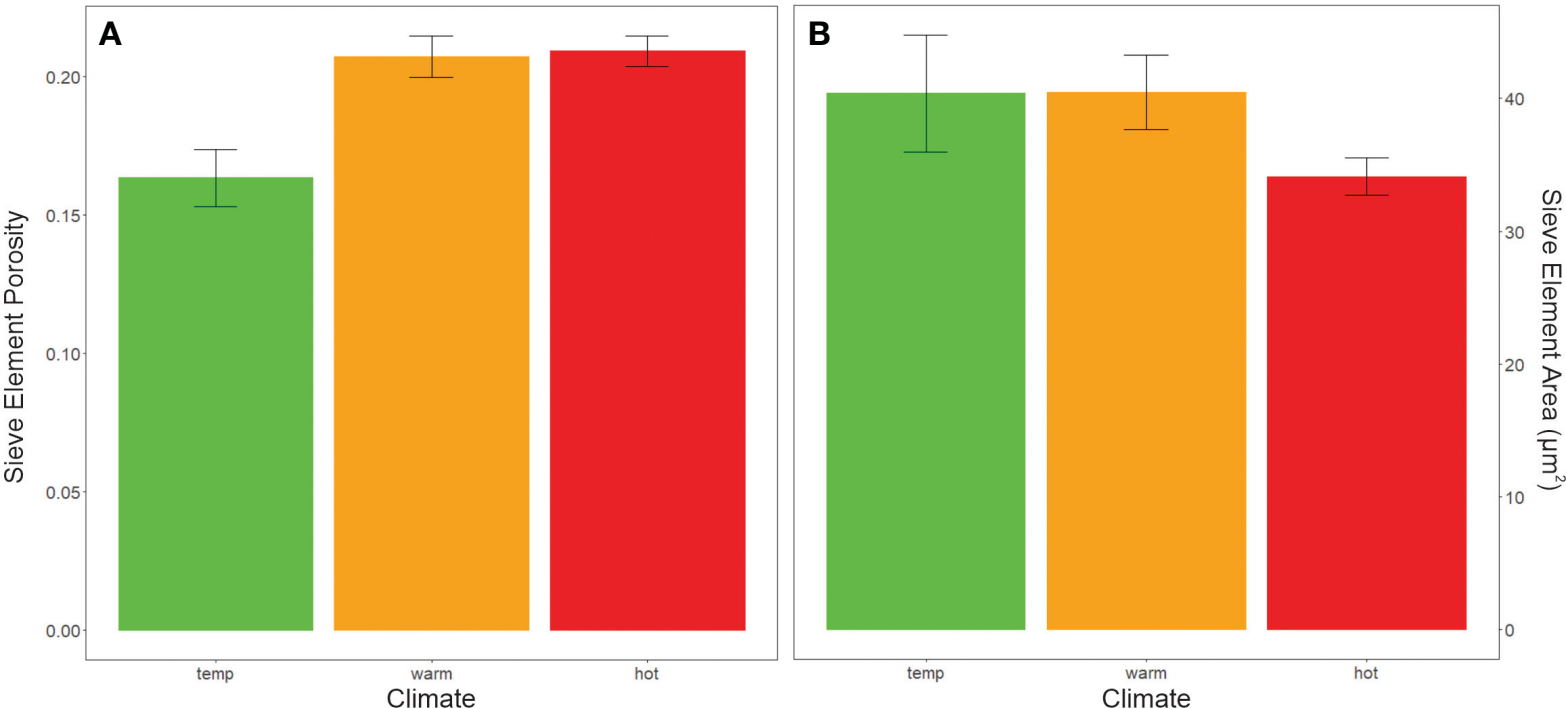
Anatomical data from sieve plates of pedicels categorized by cultivar climate categories. **(A)** Porosity (the proportion of pore area open for sap flow in a sieve plate) was greater in hot and warm climate grapes than temperate climate grapes (ANOVA, F=4.964, P < 0.05). **(B)** however, sieve element area did not significantly differ between climate groups.

Sieve plate porosity was also significantly different between climate groups. Porosity was significantly lower (i.e., plates were less open) in cultivars typically grown in temperate than hot or warm regions (ANOVA, F=4.964, P< 0.05, **Figure 7A**). In contrast, mean sieve element areas did not differ between climate or color groups (ANOVA, F= 1.45, P ≥ 0.05, **Figure 7B**).

### Total sieve element resistances are much higher in pedicels than in stems

Compared to past work on sieve element resistances in *V. vinifera*, we found that pedicel total sieve element resistances nearly 3 orders of magnitude greater than in stem sieve elements (**Table 4**). Interestingly, in stem sieve elements, the contribution towards resistance from the sieve plate and sieve element lumen is split almost equally. However, in our pedicel samples, sieve plate resistance accounts for between 66-87% of the total pathway resistance. This is mostly due to sieve elements in stems being ~9.3x times longer than sieve elements in pedicels, thus increasing the relative contribution of the sieve plate to total resistance over that of the lumen in pedicels.

**Table 4:** Descriptive statistics and phloem sieve element resistance of selected red and white grape cultivars.

Cultivar	Grape Color	Climate	r (µm)	l (µm)	r_p_ (µm)	l_p_ (µm)	N_p_	R_lumen_ resistance (%)	R_plate_ resistance (%)	R_SE_ (Pa * s/µm^2^)
Syrah	red	hot	2.87 +/- 0.41	54.2 +/- 13.2	0.22 +/- 0.18	0.37 +/- 0.22	23.6 +/- 8.38	34.4	65.6	1.21e16
Barbera	red	warm	3.72 +/- 0.86	60.6 +/- 13.3	0.25 +/- 0.09	0.36 +/- 0.09	22.2 +/- 4.92	33.7	66.3	1.45e16
Fiano	white	hot	3.08 +/- 0.53	44.6 +/- 13.2	0.22 +/- 0.07	0.25 +/- 0.07	24.4 +/- 6.67	11.2	88.8	1.95e16
Verdelho	white	hot	3.27 +/- 0.85	54.9 +/- 13.3	0.19 +/- 0.19	0.26 +/- 0.04	25.4 +/- 9.63	13.0	87.0	2.86e16
*V. vinifera* from Liesche et al., 2017 stems	–	–	18 +/- 4	500 +/- 100	0.7 +/- 0.23	3.5	661 +/- 64	49.5	50.5	4.8e13

For each sieve element character, numbers represent means +/- standard deviation. = r (sieve element radius), l (sieve element length), r_p_ (sieve pore radius), l_p_ (sieve plate thickness), N_p_ (number of sieve plate pores). As a comparison, data from stem sieve element characteristics of *V. vinifera* stem organs are included from Liesche et al. (2017).

### Phloem traits are stronger predictors of sugar accumulation than vine water and carbon status

Phloem anatomy was a stronger predictor of maximum sugar accumulation rates than vine carbon gain or water stress. Maximum sugar accumulation rates were not significantly correlated with photosynthesis (r^2^< 0.03, P ≥ 0.05, N = 14) or midday leaf water potentials (r^2^ = 0.05, P ≥ 0.05, N = 14). Including photosynthesis and midday leaf water potential as additional predictors also did not substantively improve the relationships between maximum sugar accumulation rates and petiole or pedicel cross-sectional phloem areas. Akaike Information Criterion corrected for small sample size (AICc) values were higher for the larger models than the univariate models predicting maximum accumulation rates from petiole or pedicel phloem area alone, indicating that accounting for vine carbon gain and water stress did not improve predictive capacity for sugar accumulation (**Table 2**). In addition, only one correlation was found between phloem petiole area and minimum mid-day water potential (r =-0.506, p<0.05), while other average photosynthesis and water potential variables did not correlate with the phloem anatomical parameters. Finally, a previous dataset measuring leaf area for each cultivar did not find any significant correlations with °Brix accumulation, or other parameters measured (see **Supplementary Table S2** for correlation matrix p-values of all variables measured in study).

## Discussion

Overall, we found that total cross-sectional phloem area in the pedicels and the petioles significantly predicted maximum °Brix accumulation rates in the berries (**Figure 3**), as well as sieve element area in pedicels (**Figure 5**). Other sieve tube traits, such as sieve plate porosity, were not correlated with sugar accumulation rates, indicating that grapevines mainly increase their maximum capacity for sugar transport by adding more and wider sieve tubes to the transport pathway. Total cross-sectional areas were significantly lower in cultivars typically grown in hot than warm growing regions, suggesting these cultivars have been inadvertently selected for smaller phloem areas to slow sugar accumulation, delay ripening, and achieve an optimal flavor profile provided by longer grape maturation times prior to harvest (**Figure 6**). Further, although there wasn’t a significant difference in sieve element area between cultivar climate category in the pedicel phloem, sieve element area did significantly predict brix accumulation rate. Phloem area was also a stronger predictor for sugar accumulation rates than the typical vegetative physiology parameters of gas exchange and water potential (**Table 2**). This study points to a new anatomical phenotype that can be used by grape breeders to select for cultivars with smaller petiole or pedicel phloem areas to decrease sugar accumulation rates to berries as an adaptation to increasing temperature.

### Phloem area as a predictor of sugar accumulation in grape berries

Our phloem area and °Brix accumulation results align with findings from trait comparisons in other crop species and experiments manipulating phloem area in grape and other crops. In grapevine (Malbec), abscisic acid and gibberellin hormone treatments increased the phloem cross-sectional area in the midveins, pedicels, and stems along with berry sugar concentrations, despite reduced photosynthetic assimilation (Murcia et al., 2016). The increased phloem area enhances the hydraulic conductivity of the transport pathway (Savage et al., 2015), facilitating the transport of sugars from source to sink (Hölttä et al., 2009). Phloem area has also been linked to fruit growth and sugar accumulation in other crop species. For example, modifying the expression of a phloem cell proliferation regulatory gene in tomato increased phloem area, yield, and fruit sugar concentration (Nam et al., 2022). Similarly, in giant pumpkin varieties, the phloem area in pedicels and petioles was positively correlated with fruit yield (Savage et al., 2015). These findings highlight the potential for optimizing phloem area to enhance plant productivity by matching source production and sink utilization. Additionally, our study suggests that targeting phloem/xylem in petioles could be an efficient approach for plant breeders to improve yield by enhancing hydraulic conductance and carbon export to fruits (Brocious and Hacke, 2016).

### Cultivar by climate sugar accumulation patterns and linkage to vascular area

One of the goals of this study was to investigate how cultivars adapted to different climate regimes varied in sugar accumulation and vascular anatomy traits under common garden conditions. Approximately half of the variance in berry sugar concentration is attributable to climate (Suter et al., 2021), making common garden experiments crucial to isolate the effects of plant traits on sugar accumulation. We found that, for red varieties, total phloem cross-sectional area in the petioles and pedicels was significantly larger in the varieties typically grown in warm regions than hot regions (**Figure 6**) (Average growing season temperature ranges from 17 – 19°C for warm regions and >19°C for hot regions). This could be an adaptation unknowingly selected by generations of winemakers to slow sugar accumulation and synchronize sugar and flavor development in hot climates. For white varieties, phloem area did not increase significantly from hot to warm regions (**Figure 6**). There could have been less selective pressure to increase sugar accumulation in the warm-climate white than red varieties, since white wines are typically made with lower alcohol content, and the absence of anthocyanin production could reduce metabolic demands for sugar (Bogs et al., 2006; Deloire, 2013).

Phloem anatomy is influenced by both the climate that plants have adapted to and the climate plants experience during the growing season (i.e., plasticity). This suggests that more work is needed to evaluate how plastic responses to interannual or geographic variability to climate influence cultivar differences in phloem anatomy and sugar transport capacity. In *Arabidopsis*, the effects of growing conditions on phloem anatomy depended strongly on the climate the genotypes evolved in. Comparisons between cool and hot growing conditions showed that high temperatures reduced the proportion of phloem area in the minor veins, and that these reductions were larger in *Arabidopsis* genotypes that evolved in cool than hot climates (Adams et al., 2016; Stewart et al., 2017). These results suggested that phloem plasticity in response to growing conditions outside evolved temperature ranges was greater in genotypes adapted to cool climates, increasing genotypic differences in phloem anatomy under hot growing conditions. Interestingly, we found the opposite pattern in grape, that phloem area in the pedicel and petiole was significantly larger in the cultivars typically grown in warm than hot climates, even though our common garden experiment was in a hot growing region (i.e., with a mean temperature of 20.1°C over the 2020 growing season). Comparisons in different regions or in years with different climatic conditions are needed to determine how strongly the cultivar differences in anatomy observed here depend on the conditions during phloem development.

### Effects of xylem and phloem scaling on °Brix accumulation

Xylem and phloem area scaled in the midvein, petiole, and pedicel, which produced similar relationships in xylem and phloem areas with maximum °Brix accumulation rates and climate groupings (**Figure 4**). The relationships with xylem area could simply reflect selection for phloem traits and developmental constraints that make xylem and phloem differentiation proportional, or both xylem and phloem area could impact °Brix accumulation rates. °Brix is a concentration and determined by water and sugar contents. The phloem supplies most (> 80%) of the water to the berries after veraison (Coombe and McMarthy, 2000; Keller et al., 2006). The total volume of phloem water influx is generally much larger than the volume of the berries, forcing the berries to export water to the canopy through the xylem to avoid cracking or splitting (Matthews and Shackel, 2005; Keller et al., 2015). A larger phloem area would increase the water influx into the berries, which could require a larger xylem area to compensate for water export. Further, the xylem accounted for most of the vascular area in each organ, and the ratio of xylem to phloem area increased with stem cross sectional area, which also made this ratio significantly larger in warm- than hot-climate cultivars (**Figures 4**, **6**). This larger xylem:phloem ratio could accelerate °Brix accumulation by increasing the capacity for water export relative to influx. Thus, selecting for a lower xylem:phloem ratio could slow berry sugar accumulation. Xylem and phloem areas also scale in other species, including ash (Kiorapostolou and Petit, 2019), *Pelargonium* (Ray and Jones, 2018), fir (Zhang et al., 2021), poplar, and ginkgo (Carvalho et al., 2018), and, notably, xylem:phloem ratios were smaller in species with larger fruit (i.e., the phloem accounted for 57% of pedicel vascular area in tomato and 39% in grape) (Simon et al., 2022). However, some grape cultivars produce blockages in the pedicel xylem during ripening (Choat et al., 2009; Knipfer et al., 2015) that reduce conductivity and water efflux, which could make the ratio of xylem to phloem area less important to °Brix accumulation rates. Overall, more work is needed to clarify the effects of individual tissue areas and xylem:phloem area ratios on ripening.

### Sieve tube characteristics did not differ between warm and hot climate cultivars

Another interesting findings from the current study was that sieve element area was a significant predictor of brix accumulation rate (**Figure 5**), while porosity of the elements (openness of sieve plate was not. Although sieve element area did not significantly differ by climate grouping (**Figure 7**), most of the smaller sieve element area/lower brix accumulation rate cultivars were from the warm climate category. These findings suggest that grapevines have primarily adapted to control sugar accumulation rate by changing the number and width of sieve elements, although these two traits were not correlated (**Supplementary Table S2**). Conversely, phloem cross-sectional area and mean sieve element area were correlated in the pedicels for other species, including pumpkin (Savage et al., 2015) and tomato (Bussières, 2002). However, similar to our findings, variation in sieve element area was small for pumpkin, and the differences between cultivars were not significant (Savage et al., 2015). Sieve plate porosity (the openness for flow) was significantly lower in the temperate-climate cultivars (**Figure 7A**) (i.e., typical grown in a mean growing season temperature from 15 - 17°C). Cooler growing regions are typically more humid and prone to disease pressure (Caffarra et al., 2012), and less porous sieve plates can facilitate the faster formation of callose blockages to more quickly restrict pathogen spread through the phloem (Miller et al., 2021). Future work may consider the transcriptional abundance of sugar unloading proteins (e.g. Chen et al., 2012), and how this relates with phloem anatomical characteristics related to pathway resistance.

### Water relations and photosynthesis is less predictive of °Brix accumulation than phloem area

Phloem anatomy was a stronger predictor of berry sugar accumulation rates than vegetative physiology parameters capturing vine carbon gain and water status. This was unexpected, since photosynthesis determines the carbon available for ripening, and water stress has been shown to strongly impact sugar accumulation rates in many of the cultivars tested here (Gambetta et al., 2020; Suter et al., 2021). However, our experimental vines were irrigated during the ripening period to maintain leaf water potentials in a relatively narrow range (i.e., -1.71 – -0.016 MPa). This irrigation regime follows standard commercial practices for California, which could have limited cultivar differences in vine water stress and photosynthesis and thus, their impacts on sugar accumulation. These findings suggest that measuring phloem anatomy could provide more insight into plant capacity for berry sugar accumulation under standard, irrigated conditions than conventional vegetative physiology traits.

Alternatively, leaf-level photosynthesis could have been decoupled from °Brix accumulation by variation in vine balance (i.e., the ratio of canopy area to fruit mass), which would impact the ratio of whole-plant carbon supply to demand. A larger ratio of canopy area to fruit mass would increase maximum °Brix accumulation rates. Future work should estimate leaf area per cultivar to ensure that relationships between phloem anatomy and maximum °Brix accumulation rates scale with variation in vine balance.

### Conclusions and future work

Overall, we found that phloem cross-sectional area in the petioles and pedicels was the most predictive trait for the maximum rate of sugar accumulation in the berries across winegrape cultivars tested. Carbon dioxide in the atmosphere is expected to double by centuries’ end, and the dual effects on plant carbon availability and growing season temperature are projected to strongly accelerate sugar accumulation and exacerbate the detrimental impacts on wine quality (Alston et al., 2011; Bigard et al., 2018; Delrot et al., 2020). We suggest that reduced phloem areas could be a useful and novel phenotype to screen for in existing cultivars to slow carbon transport rates in hotter growing regions, allowing more time for flavor development. If petiole phloem area is well-conserved across life stages and under different growing conditions, this would be an especially useful trait to accelerate phenotyping since grapevines must mature for several years to begin producing fruit. However, future work is still needed to clarify how xylem area and vine balance (i.e., whole-plant source:sink ratios) influence sugar concentrations, and how interannual and site-specific environmental variability influence anatomical traits and sugar accumulation.

## Data availability statement

The original contributions presented in the study are included in the article/**Supplementary Material**. Further inquiries can be directed to the corresponding author.

## Author contributions

RS: Conceptualization, Formal analysis, Funding acquisition, Investigation, Methodology, Project administration, Resources, Supervision, Validation, Visualization, Writing – original draft, Writing – review & editing. EF: Conceptualization, Data curation, Formal analysis, Funding acquisition, Investigation, Methodology, Project administration, Resources, Supervision, Validation, Writing – original draft, Writing – review & editing. KE: Data curation, Methodology, Writing – original draft, Writing – review & editing, Formal analysis. SB: Data curation, Formal analysis, Methodology, Writing – original draft, Writing – review & editing. MB: Conceptualization, Data curation, Formal analysis, Funding acquisition, Investigation, Methodology, Project administration, Resources, Supervision, Validation, Writing – original draft, Writing – review & editing.
