# Supplementary material for: Phloem anatomy predicts berry sugar accumulation across 13 wine-grape cultivars
Source: Front Plant Sci. 2024 Mar 21;15:1360381. doi: 10.3389/fpls.2024.1360381 (PMC10991835; doi:10.3389/fpls.2024.1360381)
Supplement: Supplementary file 1 [file DataSheet_1.zip › Figure captions.docx]

Supplemental Figure Legends

Figure S1. High and low daily temperatures from the Davis CIMIS Station. The black line tracks temperature ranges throughout the day the red line show the overall average temperature per day. The time between vertical dotted lines indicate the study period.

Figure S2. The ⁰Brix accumulation per growing degree day for 13 cultivars under investigation in this study that fit a logistic model of ⁰Brix accumulation.

Figure S3. Maximum Δ ⁰Brix accumulation per growing degree day as a function of the ratio of phloem cross sectional area: total sectional area. This was a measure of if the overall cross-sectional area could be used as a predictor of sugar accumulation rate. Organs included are the midvein A) Pedicel, B) Petiole and C) Midvein. Only the petiole had a significant relationship between Maximum Δ ⁰Brix accumulation and phloem area: total cross-sectional area.

Figure S4. The overall petiole area did not predict Maximum Δ ⁰Brix accumulation.
