# Supplementary material for: Phloem anatomy predicts berry sugar accumulation across 13 wine-grape cultivars
Source: Front Plant Sci. 2024 Mar 21;15:1360381. doi: 10.3389/fpls.2024.1360381 (PMC10991835; doi:10.3389/fpls.2024.1360381)
Supplement: Supplementary file 1 [file DataSheet_1.zip › Table S1.docx]

| Cultivar | Vine Location Code |
| --- | --- |
| Chardonnay | A2R1V1 |
| Chardonnay | A2R1V2 |
| Riesling | A2R2V12 |
| Riesling | A2R2V13 |
| Zinfandel | A2R4V9 |
| Zinfandel | A2R4V10 |
| Syrah | A2R5V16 |
| Syrah | A2R5V15 |
| Sangiovese | A2R5V4 |
| Sangiovese | A2R5V5 |
| Merlot | A2R6V1 |
| Merlot | A2R6V2 |
| Pinot noir | A2R7V11 |
| Pinot noir | A2R7V12 |
| Tempranillo | A2R8V1 |
| Tempranillo | A2R8V2 |
| Nebbiolo | A2R10V4 |
| Nebbiolo | A2R10V5 |
| Carignan | A1R2V1 |
| Carignan | A1R2V2 |
| Barbera | A1R2V22 |
| Barbera | A1R2V23 |
| Cabernet Sauvignon | A1R2V46 |
| Cabernet Sauvignon | A1R2V47 |
| Aglianico | A1R3V46 |
| Aglianico | A1R3V47 |
| Montepulciano | A1R3V43 |
| Montepulciano | A1R3V44 |
| Fiano | A1R3V34 |
| Fiano | A1R3V35 |
| Verdello | A1R3V28 |
| Verdello | A1R3V29 |
| Mourredre | A2R8V14 |
| Sauvignon Blanc | A1R1V14 |
| Sauvignon Blanc | A1R1V13 |

Supplemental Table S1. Grapevine (*V. vinifera*) cultivars and their location code at the Robert Mondavi Teaching Vineyard on the UC Davis Campus. Code key: A1 = Student Rotation Block, A2 = Ampelography Block, R = Row Number, V = Vine Number.
