# Supplementary figures and images for: Phloem anatomy predicts berry sugar accumulation across 13 wine-grape cultivars
Source: Front Plant Sci. 2024 Mar 21;15:1360381. doi: 10.3389/fpls.2024.1360381 (PMC10991835; doi:10.3389/fpls.2024.1360381)

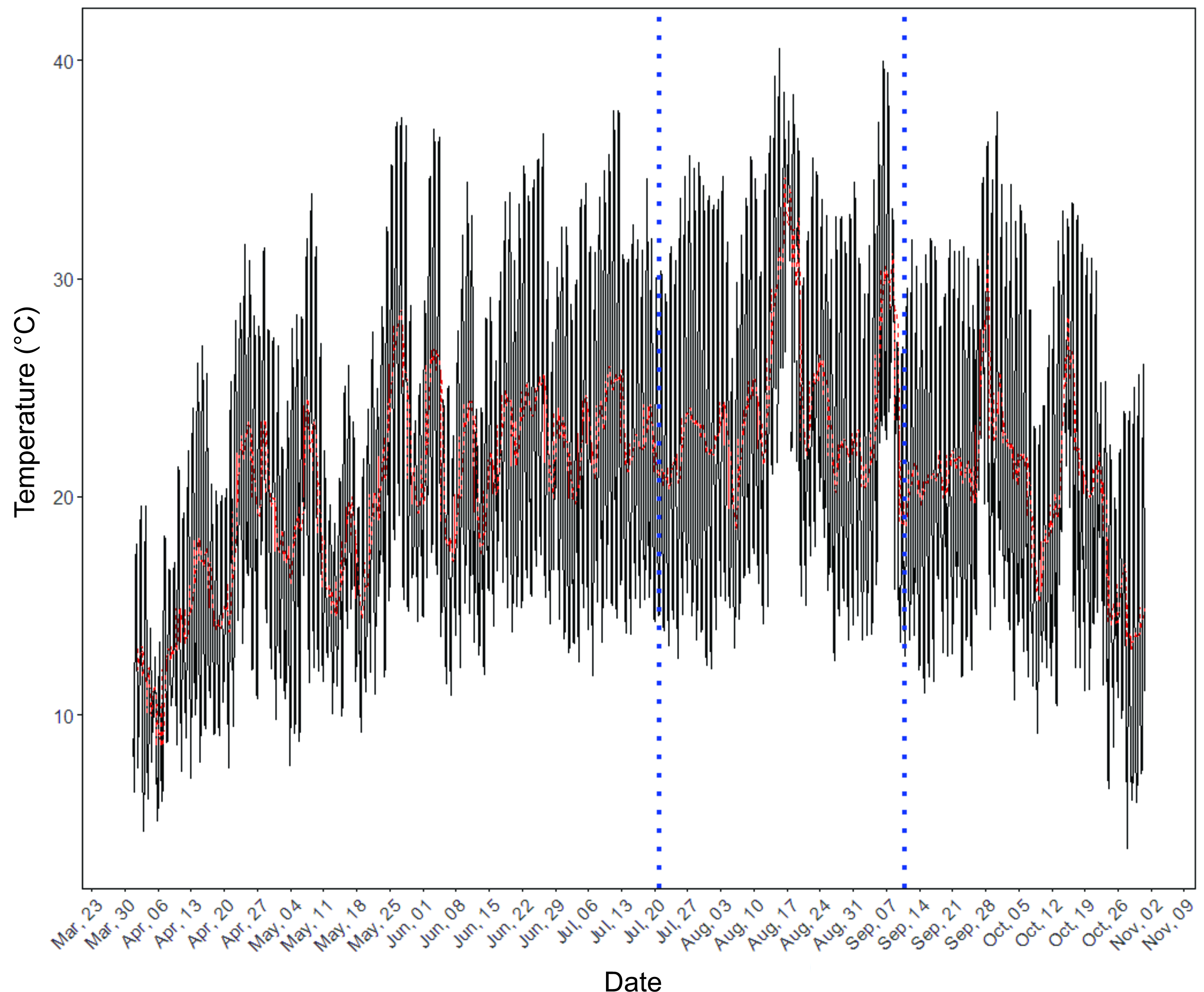

Supplement: Supplementary file 1 [file DataSheet_1.zip › Figure S1.TIF]

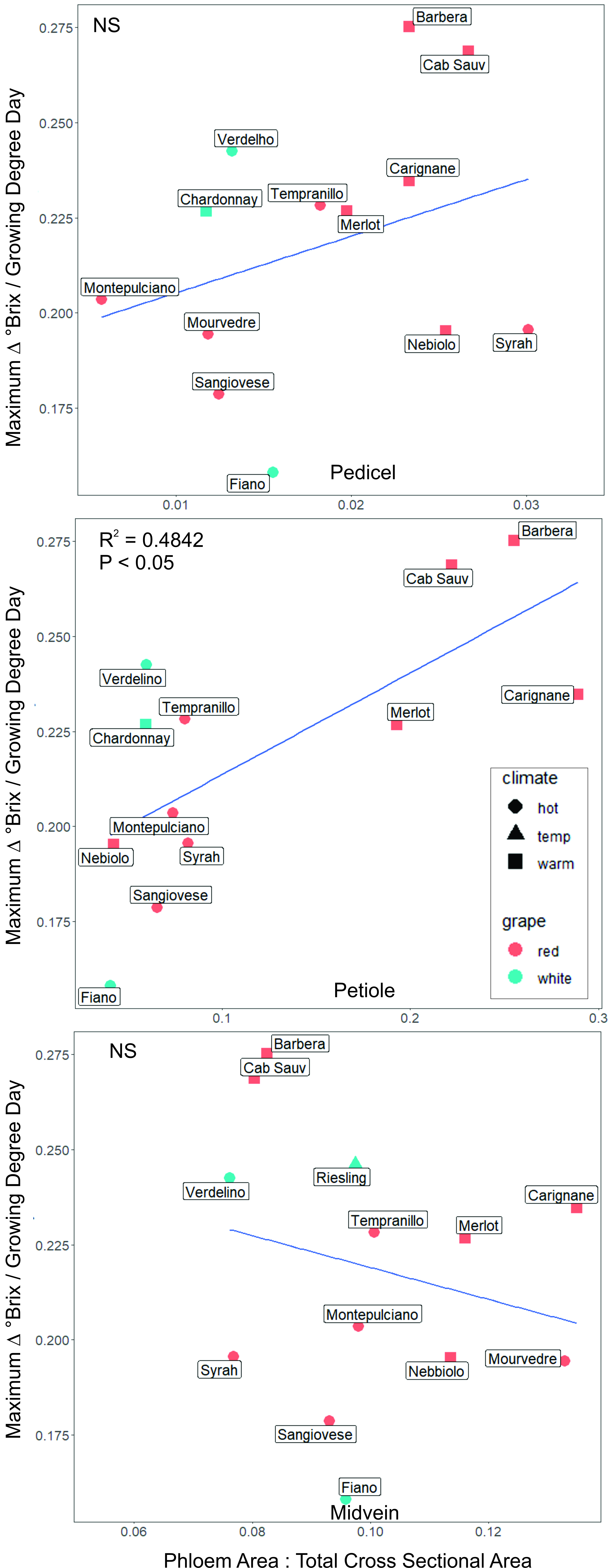

Supplement: Supplementary file 1 [file DataSheet_1.zip › Figure S3.JPEG]

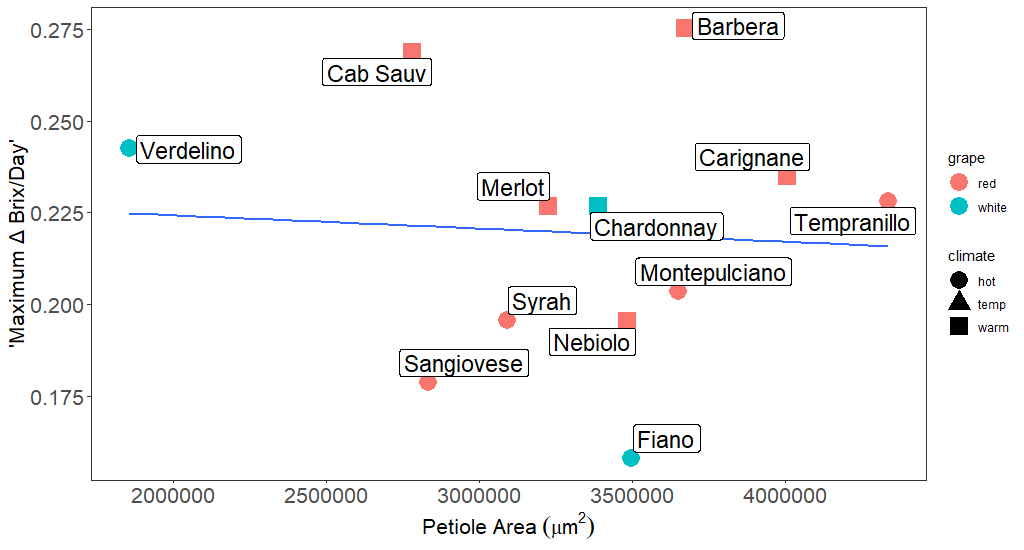

Supplement: Supplementary file 1 [file DataSheet_1.zip › Figure S4.TIFF]
